# The Anti-diabetic Drug Gliquidone Modulates Lipopolysaccharide-Mediated Microglial Neuroinflammatory Responses by Inhibiting the NLRP3 Inflammasome

**DOI:** 10.3389/fnagi.2021.754123

**Published:** 2021-10-29

**Authors:** Jieun Kim, Jin-Hee Park, Keshvi Shah, Scott John Mitchell, Kwangwook Cho, Hyang-Sook Hoe

**Affiliations:** ^1^Department of Neural Development and Disease, Korea Brain Research Institute (KBRI), Daegu, South Korea; ^2^UK-Dementia Research Institute, Department of Basic and Clinical Neuroscience, Institute of Psychiatry, Psychology and Neuroscience, King’s College London, London, United Kingdom; ^3^Department of Brain and Cognitive Sciences, Daegu Gyeongbuk Institute of Science & Technology (DGIST), Daegu, South Korea

**Keywords:** neuroinflammation, microgliosis, LPS (lipopolysaccharide), gliquidone, proinflammatory cytokine, NLRP3 inflammasome

## Abstract

The sulfonylurea drug gliquidone is FDA approved for the treatment of type 2 diabetes. Binding of gliquidone to ATP-sensitive potassium channels (SUR1, Kir6 subunit) in pancreatic β-cells increases insulin release to regulate blood glucose levels. Diabetes has been associated with increased levels of neuroinflammation, and therefore the potential effects of gliquidone on micro- and astroglial neuroinflammatory responses in the brain are of interest. Here, we found that gliquidone suppressed LPS-mediated microgliosis, microglial hypertrophy, and proinflammatory cytokine COX-2 and IL-6 levels in wild-type mice, with smaller effects on astrogliosis. Importantly, gliquidone downregulated the LPS-induced microglial NLRP3 inflammasome and peripheral inflammation in wild-type mice. An investigation of the molecular mechanism of the effects of gliquidone on LPS-stimulated proinflammatory responses showed that in BV2 microglial cells, gliquidone significantly decreased LPS-induced proinflammatory cytokine levels and inhibited ERK/STAT3/NF-κB phosphorylation by altering NLRP3 inflammasome activation. In primary astrocytes, gliquidone selectively affected LPS-mediated proinflammatory cytokine expression and decreased STAT3/NF-κB signaling in an NLRP3-independent manner. These results indicate that gliquidone differentially modulates LPS-induced microglial and astroglial neuroinflammation in BV2 microglial cells, primary astrocytes, and a model of neuroinflammatory disease.

## Introduction

Diabetes mellitus is a serious metabolic disorder characterized by elevated blood glucose levels (hyperglycemia) due to improper insulin function (Ramos-Rodriguez et al., 2014). Long-term hyperglycemia can impair macrovascular and microvascular function and cause brain atrophy, neuropathy, and neuroinflammation (Wrighten et al., 2009; Ramos-Rodriguez et al., 2014). Moreover, type 2 diabetes and insulin resistance can lead to peripheral meta-inflammation and an overproduction of proinflammatory cytokines (Ferreira et al., 2014). Clusters of proteins that are differentially regulated in type 2 diabetes have also been linked to neuroinflammation and related neurodegenerative diseases, including Alzheimer’s disease (AD) (Mittal et al., 2016). Clinical studies have shown that some classes of diabetes medications can rescue both glycemic control and inflammation-linked deficits, providing benefits for cognitive function and reducing dementia risk (Chatterjee and Mudher, 2018; Weinstein et al., 2019; Akimoto et al., 2020). However, the molecular mechanisms by which diabetes medications affect neuroinflammatory responses have not been fully elucidated.

Therapeutic interventions for type 2 diabetes include the class of drugs known as sulfonylureas, such as gliquidone and glibenclamide. These drugs bind the sulfonylurea receptor on the surface of pancreatic β-cells, which functions as a subunit of ATP-sensitive potassium (K-ATP) channels (Aquilante, 2010; Sola et al., 2015). Gliquidone inhibits K^+^ efflux in the cell membrane, resulting in depolarization, activation of voltage-gated calcium channels and calcium influx (de Wet and Proks, 2015). The resulting increase in calcium conductance leads to insulin expression and release (Yang and Berggren, 2006). Isotope tracing studies using [^3^H]glibenclamide ([^3^H]Grb) have demonstrated that glibenclamide crosses the blood-brain barrier (BBB) in wild-type mice. In addition, glibenclamide reaches the brain more easily after cerebral ischemic insult (Angel and Bidet, 1991; Ortega et al., 2012). As further evidence that sulfonylurea drugs can cross the BBB, gliquidone injection decreases the immobility time of mice in the forced swimming test (Galeotti et al., 1999), and gliquidone significantly decreases cromakalim-evoked potassium efflux in slices of the rat substantia nigra (Schmid-Antomarchi et al., 1990). These observations suggest that the sulfonylurea family of type 2 diabetes drugs may provide additional potential therapeutic benefits by reducing insulin-mediated neuroinflammation.

As part of the neuroinflammatory response, NLRP3/IL-1β/caspase-1 inflammasome formation is promoted. NLRP3 is one of four known NLRP inflammasome types, named Nod-like receptor (NLRP) 1 to 4 (Franchi et al., 2009). NLRP3 is dominantly involved in neuroinflammatory responses *in vitro* and *in vivo*. For instance, NLRP3 activates TLR4 signaling, which leads to the induction of neuroinflammatory responses in an NF-κB-dependent manner (Paik et al., 2021). Interestingly, K^+^-channel agonists (e.g., Leu-Leu-*O*-methyl ester) and/or Kir6.2 siRNA have been reported to modulate neuroinflammatory responses through the NLRP3 inflammasome (Munoz-Planillo et al., 2013; Qu et al., 2017; Yang et al., 2019). However, it is unclear whether gliquidone, an antagonist of K-ATP channels (specifically, the SUR1 and Kir6 subunits), modulates neuroinflammatory responses by regulating NLRP3 inflammasome activation.

To address this gap, in this study we investigated whether gliquidone affects lipopolysaccharide (LPS)-stimulated micro- and astroglial neuroinflammatory responses. We found that gliquidone suppressed LPS-induced microglial activation and proinflammatory cytokine levels by altering NLRP3 inflammasome activation in wild-type mice, with smaller effects on astrogliosis. In BV2 microglial cells, gliquidone significantly suppressed the LPS-induced increase in proinflammatory cytokine levels by altering ERK/STAT3/NF-κB signaling. Importantly, gliquidone downregulated LPS-mediated neuroinflammatory responses in BV2 microglial cells by inhibiting NLRP3 inflammasome formation. In primary astrocytes, gliquidone reduced LPS-induced neuroinflammatory responses by regulating STAT3/NF-κB signaling but not NLRP3 inflammasome activation. Taken together, these data suggest that the anti-diabetic drug gliquidone may be a new therapeutic intervention for neuroinflammation-mediated neuropathy in the brain.

## Materials and Methods

### Ethics Statement

All experiments were approved by the institutional biosafety committee (IBC) and performed in accordance with approved animal protocols of the Korea Brain Research Institute (KBRI, approval no. IACUC-19-00042).

### Gliquidone and Lipopolysaccharide

Gliquidone was purchased from Selleck Chemicals (S3151, Houston, TX, United States) and Tokyo Chemical Industry Co., Ltd. (G0332, Tokyo, Japan). Gliquidone was used at a concentration of 5 μM (in 1% DMSO) in *in vitro* assays and injected at a dose of 10 or 20 mg/kg (i.p., dissolved in 5% DMSO, 10% PEG, 20% Tween 80) in *in vivo* experiments.

### C57BL6/N Mice

Male C57BL6/N mice (wild-type; Hana Company, Busan, South Korea) were used in *in vivo* experiments. A pathogen-free facility with a 12 h light/dark cycle and a temperature of 22°C was used to house all mice. Daily injections of gliquidone (10 or 20 mg/kg) or vehicle (5% DMSO) were administered intraperitoneally (i.p.) on 3 consecutive days. On the third day, 10 mg/kg LPS (i.p.) or PBS was injected. The mice were sacrificed 8 h after the last injection and fixed in 4% paraformaldehyde. Subsequently, mouse brain slices with a thickness of 30 μm were prepared using a Leica CM1850 cryostat (Leica Biosystems, Buffalo Grove, IL, United States).

### Immunofluorescence Staining

Mouse brain slices were incubated in 10% normal goat serum (Vector Laboratories) at room temperature for 1 h, followed by immunostaining with rabbit or mouse anti-target antibody at 4°C overnight. The sections were subsequently incubated with an Alexa Fluor 488- or 555-labeled goat IgG secondary antibody for 2 h at room temperature. Sections were mounted on glass slides were covered with a DAPI (Vector Laboratories)-containing mounting solution, and images were obtained by fluorescence microscopy (DMi8, Leica Microsystems, Wetzlar, Germany) for analysis by Image J software (NIH). Detailed information on the primary and secondary antibodies used for IF and immunocytochemistry (ICC) is provided in Table 1.

**TABLE 1:** List of antibodies used for Immunofluorescence Staining (IF) or immunocytochemistry (ICC).

**Primary antibodies**						
**Immunogen**	**Host species**		**Dilution**	**Manufacturer**	**Catalog no.**	**Application**

Iba-1	Rabbit		1:500	Wako	019-19741	IF
GFAP	Rabbit		1:500	Neuromics	RA22101	IF
NLRP3	Goat		1:100	Abcam	AB4207	IF, ICC
COX-2	Rabbit		1:100	Cell Signaling	89332	IF
pERK	Rabbit		1:200	Cell Signaling	9101	ICC
IL-6	Mouse		1:50	Santa Cruz	SC-57315	IF
CD11b	Rat		1:200	Abcam	AB8878	ICC
GFAP	Chicken		1:500	Millipore	AB5541	ICC
p-STAT3^*S727*^	Rabbit		1:200	Abcam	AB86340	ICC
p-NF-κB^*S536*^	Rabbit		1:200	Cell Signaling	3033S	ICC

**Secondary antibodies**						

**Antibody**		**Dilution**		**Manufacturer**	**Catalog no.**	**Application**

Goat anti-rabbit IgG, 555		1:200		Invitrogen	A21428	IF
Goat anti-rabbit IgG, 488		1:200		Invitrogen	A11008	IF, ICC
Goat anti-mouse IgG, 488		1:200		Invitrogen	A11001	IF
Donkey anti-goat, 555		1:200		Invitrogen	A21432	IF
Goat anti-chicken IgG, 488		1:200		Abcam	A150169	IF
Goat anti-rat IgG, FITC		1:200		Invitrogen	A18866	ICC

### Enzyme-Linked Immunosorbent Assay

Wild-type mice were injected daily with gliquidone (10 or 20 mg/kg, i.p.) or vehicle (5% DMSO + 10% PEG + 20% Tween80) for 3 days, followed by injection of LPS (10 mg/kg, i.p.) or PBS 30 min after the last injection on day 3. Eight hours later, blood was sampled and centrifuged at 2,000 rpm for 20 min, and the supernatant (serum) collected and stored at −80°C until analysis. Peripheral IL-1β, IL-6, and TNF-α levels were measured using the appropriate ELISA kit (IL-1β, Cat. no. 88-7013-88; IL-6, Cat. no. 88-7064-88; and TNF-α, Cat no. 88-7324-88; Invitrogen, Waltham, MA, United States) as described by the manufacturer.

### BV2 Microglial Cell Line

BV2 microglial cells (generously provided by Dr. Kyung-Ho Suk) were cultured at 37°C and 5% CO_2_ in DMEM high glucose (Invitrogen, Carlsbad, CA, United States) containing 5% fetal bovine serum (FBS, Invitrogen).

### Mice Brain-Derived Primary Astrocyte Culture

Primary astrocytes were prepared from postnatal day 1 (P1) C57BL6/N mice as previously described (Ryu et al., 2019). Briefly, whole brains were dissected and minced through 70 μm mesh, and the mixed cells were grown in low-glucose DMEM (1,000 mg/l glucose) supplemented with 10% FBS, 100 unit/ml penicillin, and 100 μg/ml streptomycin for 2 weeks. On day 14, primary microglial cells were detached by shaking at 250 rpm at room temperature overnight, followed by dissociation of primary astrocytes using trypsin-EDTA. After 3 rounds of washing and centrifugation for 10 min at 2,000 rpm, the pellet containing primary astrocytes was used for experiments. The cells were seeded at 7.0 × 10^5^ cells/well in 12-well plates for q-PCR and 2.5 × 10^5^ cells/well for ICC. After 2 days, the primary astrocytes were fixed in 4% paraformaldehyde at room temperature for 10 min and immunostained with an antibody against GFAP (a marker of astrocytes) at 4°C overnight. Next, the cells were washed thrice with PBS, and secondary antibodies were added for 1 h. Finally, the cells were washed with PBS, incubated with DAPI, and mounted. Images were acquired by fluorescence microscopy (DMi8, Leica Microsystems, Wetzlar, Germany), and primary astrocyte purity was calculated as follows: [Astrocyte purity (%) = (GFAP- and DAPI-positive cells/DAPI-positive cells) × 100].

### Cell Counting Kit-8 (CCK-8) Assay

Cell viability and cytotoxicity were assessed with the CCK-8 assay (Dongin Biotech Co., Ltd., Seoul, South Korea) as recommended by the manufacturer. After seeding in 96-well plates at a density of 2 × 10^4^ cells/well and incubation overnight at 37°C, BV2 cells were treated with gliquidone (0.1, 1, 5, 10, 25, and 50 μM) or vehicle (0.001, 0.01, 0.05, 0.1, 0.25, and 0.5% DMSO) for 6 h at 37°C. Next, CCK solution was added and incubated in the dark for 0.5 h, followed by detection at 450 nm in a SPECTROstar Nano microplate reader (BMG Labtech, Germany).

### MTT Assay

The cytotoxicity of gliquidone was assessed using the 3-(4,5-dimethylthiazol-2-yl)-2,5-diphenyltetrazolium bromide (MTT) assay. BV2 cells seeded into 96-well plates without FBS at a density of 4 × 10^4^ cells/well were treated for 24 h with gliquidone (0.1, 1, 5, 10, 25, and 50 μM) or vehicle (0.001, 0.01, 0.05, 0.1, 0.25, and 0.5% DMSO), followed by incubation with 0.5 mg/ml MTT for 3 h in the dark. Finally, DMSO was added to dissolve the formazan crystals with shaking, and the absorbance at 570 nm was measured using a SPECTROstar Nano microplate reader (BMG Labtech, Germany).

### Reverse Transcription-Polymerase Chain Reaction

Reverse transcription-polymerase chain reaction was performed to assess the effects of gliquidone on LPS-evoked microglial and astroglial inflammatory cytokine levels. First, total RNA was isolated from BV2 microglial cells or primary astrocytes using QIAzol Lysis Reagent (Qiagen, Cat No. 79306) and reverse transcribed into cDNA. The cDNA was subsequently used as the template in RT-PCR using Prime Taq Premix (GeNet Bio) as previously described with the primers shown in Table 2. The amplicons were separated by electrophoresis on a 1.5% agarose gel containing EcoDye (1:5000, Biofact, Daejeon, South Korea), and images were analyzed using the software Fusion Capt Advance (Vilber Lourmat, Eberhardzell, Germany).

**TABLE 2:** Sequences of primers used for reverse transcription-polymerase chain reaction (RT-PCR).

**Gene name**		**Sequence**
*il-1*β	Sense	5′-AGC TGG AGA GTG TGG ATC CC-3′
	Antisense	5′-CCT GTC TTG GCC GAG GAC TA-3′
*il-6*	Sense	5′-CCA CTT CAC AAG TCG GAG GC-3′
	Antisense	5′-GGA GAG CAT TGG AAA TTG GGG T-3′
*cox-2*	Sense	5′-GCC AGC AAA GCC TAG AGC-3′
	Antisense	5′-GCC TTC TGC AGT CCA GGT TC-3′
*inos*	Sense	5′-CCG GCA AAC CCA AGG TCT AC-3′
	Antisense	5′-GCA TTT CGC TGT CTC CCC AA-3′
*gapdh*	Sense	5′-CAG GAG CGA GAC CCC ACT AA-3′
	Antisense	5′-ATC ACG CCA CAG CTT TCC AG-3′

### Real-Time PCR (q-PCR)

The effects of gliquidone on LPS-mediated microglial and astroglial NLRP3 inflammasome activation and subsequent proinflammatory cytokine production were assessed in BV2 microglial cells and mouse primary astrocytes. In the preventive/pretreatment experiments, cells were first treated for 30 min with gliquidone (5 μM) or vehicle (1% DMSO) and then with LPS (200 ng/ml) or PBS for 5.5 h or 23.5 h. In the curative/post-treatment experiments, cells were first treated for 30 min with LPS (200 ng/ml) or PBS and then with gliquidone (5 μM) or vehicle (1% DMSO) for 5.5 h or 23.5 h. Total RNA was then extracted using TRIzol (Invitrogen, Waltham, MA, United States) as recommended by the manufacturer and reverse-transcribed (1 μg) to synthesize cDNA (GeNet Bio, Chungcheongnam-do, South Korea). The cDNA was used with Fast SYBR Green Master Mix (Thermo Fisher Scientific, Waltham, MA, United States) to perform real-time q-PCR in a QuantStudio 5 Real-Time PCR System (Applied Biosystems, Thermo Fisher Scientific, Waltham, MA, United States). The cycle threshold (*C*t) values of the mRNA levels of factors related to inflammasome and inflammation were normalized to the *C*t value for *gapdh*, and the fold change relative to the vehicle-treated control was quantified. Used primers are shown in Table 3.

**TABLE 3:** Sequences of primers used for real time-PCR.

**Gene name**		**Sequence**
*il-1*β	Sense	5′-TTG ACG GAC CCC AAA AGA TG-3′
	Antisense	5′-AGG ACA GCC CAG GTC AAA G -3′
*il-6*	Sense	5′-CCA CGG CCT TCC CTA CTT C-3′
	Antisense	5′-TTG GGA GTG GTA TCC TCT GTG A-3′
*cox-2*	Sense	5′-CCA CTT CAA GGG AGT CTG GA -3′
	Antisense	5′-AGT CAT CTG CTA CGG GAG GA-3′
*inos*	Sense	5′-GGA TCT TCC CAG GCA ACC A-3′
	Antisense	5′-TCC ACA ACT CGC TCC AAG ATT-3′
*pro-il-1*β	Sense	5′- TCT TTG AAG TTG ACG GAC CC -3′
	Antisense	5′- TGA GTG ATA CTG CCT GCC TG -3′
*nlrp3*	Sense	5′-TCC ACA ATT CTG ACC CAC AA-3′
	Antisense	5′-ACC TCA CAG AGG GTC ACC AC-3′
*sur1*	Sense	5′-GGA GAG GAA AGC CCC AGA AC-3′
	Antisense	5′-GTC ATC TTC CTC GCT CTC GG-3′
*gapdh*	Sense	5′-TGG GCT ACA CTG AGG ACC ACT-3′
	Antisense	5′-GGG AGT GTC TGT TGA AGT CG-3′

### Western Blotting

To determine the effects of gliquidone on LPS-induced ERK signaling, western blotting was conducted. BV2 cells or primary astrocytes were lysed using lysis buffer (ProPrep, iNtRON Biotechnology, Inc., Seongnam, South Korea), followed by centrifugation at 12,000 rpm for 15 min. After quantifying the protein concentration in the supernatant, 15 μg of protein sample was separated by 8% SDS gel electrophoresis. The proteins in the gel were subsequently transferred to a polyvinylidene difluoride (PVDF) membrane. After blocking with 5% skim milk or 5% BSA at room temperature for 1 h, the membrane was incubated with anti-p-ERK (1:1000, Cell Signaling), anti-ERK (1:1000, Cell Signaling), or β-actin (1:1000, Santa Cruz Biotechnology) at 4°C. Finally, HRP-conjugated goat anti-mouse IgG or HRP-conjugated goat anti-rabbit IgG (both 1:1000, Enzo Life Sciences, Farmingdale, NY, United States) was added to the membrane and incubated for 1 h, and detection was performed using ECL Reagent (GE Healthcare, Chicago, IL, United States). Fusion Capt Advance software was used for image acquisition and analysis (Vilber Lourmat).

### Immunocytochemistry

BV2 microglial cells and primary astrocytes were fixed in 4% paraformaldehyde for 10 min, washed thrice with PBS, and incubated overnight with anti-target antibody in GDB buffer as described previously (Nam et al., 2018). After washing with PBS, the cells were incubated for 1 h with an Alexa Fluor 488- or 555-conjugated antibody at room temperature. Finally, images of cells mounted in DAPI (Vector Laboratories, CA, United States) were captured using a fluorescence microscope (DMi8, Leica Microsystems, Wetzlar, Germany) and analyzed using ImageJ software.

### Statistical Analyses

GraphPad Prism 7 software (GraphPad Software, San Diego, CA, United States) was used for data analyses. Comparisons between two groups were performed using the unpaired two-tailed *T*-test with Welch’s correction; one-way ANOVA was employed for multiple comparisons (except for hippocampal NLRP3 IF). Tukey’s test or the Newman–Keuls multiple comparisons test was used for multiple comparisons with significance set at *p* < 0.05. Means ± SD are presented (^∗^*p* < 0.05, ^∗∗^*p* < 0.01, ^∗∗∗^*p* < 0.001).

## Results

### Gliquidone Decreases Lipopolysaccharide-Stimulated Microgliosis and Hypertrophy in a Model of Neuroinflammatory Disease

**FIGURE 1:**
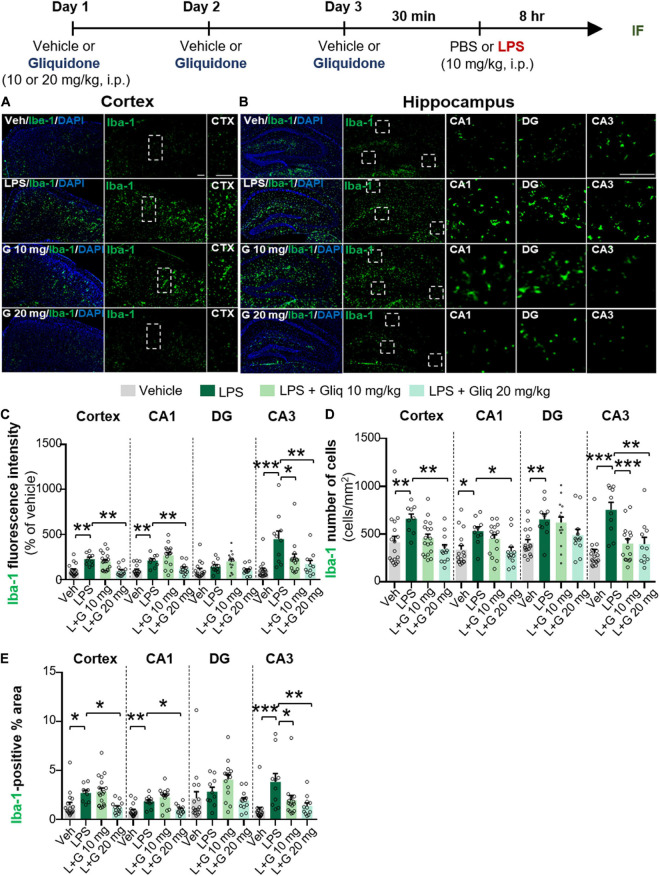
Gliquidone decreases LPS-stimulated microgliosis and hypertrophy in the cortex and hippocampus in C57BL6/N mice. The *in vivo* experimental procedure for gliquidone and LPS injection is shown at the top of the figure. **(A,B)** Immunofluorescence staining of Iba-1 expression in brain slices from mice treated as described in the experimental procedure. **(C–E)** Quantification of the results in **(A,B)** (*n* = 10–18 brain slices from 4 mice/group). *p < 0.05, **p < 0.01, ***p < 0.001. Scale bar = 100 μM.

The anti-diabetic drug gliquidone was recently shown to promote a beneficial microglial phenotype via the Kir6-containing ATP-dependent potassium channel in a mouse model of Parkinson’s disease (PD) (Du et al., 2018). However, the effects of gliquidone on LPS-induced proinflammatory responses are unknown. Therefore, we investigated whether gliquidone modulates LPS-mediated microglial activity by injecting wild-type mice daily with gliquidone (10 or 20 mg/kg, i.p.) or vehicle for 3 days; after the final administration of gliquidone, LPS (10 mg/kg, i.p.) or PBS was injected. Eight hours after LPS or PBS injection, IF staining of brain slices was performed with an anti-Iba-1 antibody, and Iba-1 intensity, Iba-1-positive cells, and Iba-1-labeled area were analyzed in the cortex and hippocampus (Figures 1A–E). Treatment with 20 mg/kg gliquidone significantly decreased the LPS-induced increases in Iba-1 immunoreactivity, Iba-1-positive cells, and Iba-1-labeled area in the cortex and hippocampal CA1 and CA3 regions (Figures 1A–E). By contrast, administration of 10 mg/kg gliquidone significantly reduced the LPS-induced increases in Iba-1 intensity, Iba-1-positive cells, and Iba-1-labeled area only in the hippocampal CA3 region (Figures 1C–E). These results indicated that a gliquidone dose of 20 mg/kg more effectively downregulates LPS-mediated microgliosis and changes in microglial kinetics and morphology in the wild-type mouse brain.

**FIGURE 2:**
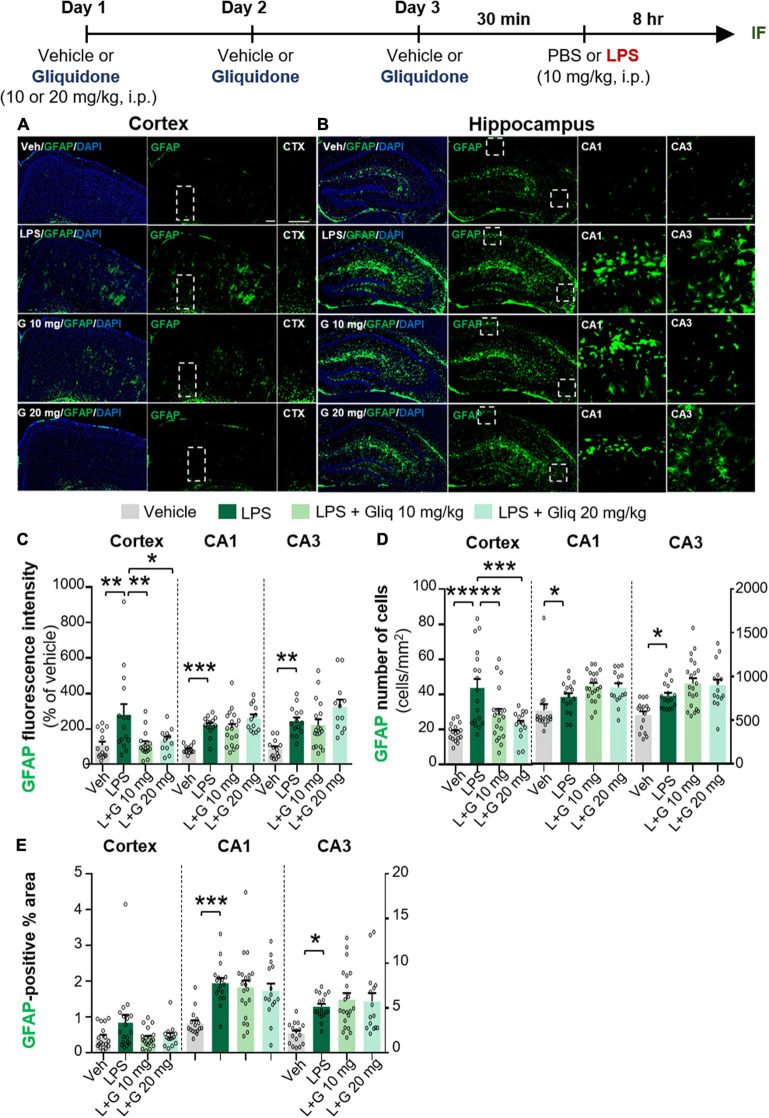
Gliquidone decreases LPS-stimulated astrogliosis and the number of GFAP-positive cells in the cortex in C57BL6/N mice. **(A,B)** Immunofluorescence staining of GFAP expression in brain slices from mice treated as shown at the top of the figure. **(C–E)** Quantification of the results in **(A,B)** (*n* = 12–18 brain slices from 4 mice/group). *p < 0.05, **p < 0.01, ***p < 0.001. Scale bar = 100 μM.

To determine if gliquidone alters LPS-evoked astrocyte activation *in vivo*, we treated wild-type mice according to same paradigm described above and performed IF staining of brain slices with an anti-GFAP antibody. GFAP intensity, GFAP-positive cells, and GFAP-labeled area were measured (Figures 2A–E). At doses of 10 and 20 mg/kg, gliquidone significantly reduced the LPS-induced increases in GFAP immunointensity and GFAP-positive cells in the cortex but not the hippocampus (Figures 2C–E). In addition, administration of 10 or 20 mg/kg gliquidone did not alter GFAP-labeled area in the cortex and hippocampus (Figure 2E). These data indicate that gliquidone modulates LPS-stimulated astrocyte activation in the mouse brain, albeit less effectively than microglial activation.

### Gliquidone Eliminates Lipopolysaccharide-Induced Increases in COX-2 and IL-6 Proinflammatory Cytokine Levels in Wild-Type Mice

**FIGURE 3:**
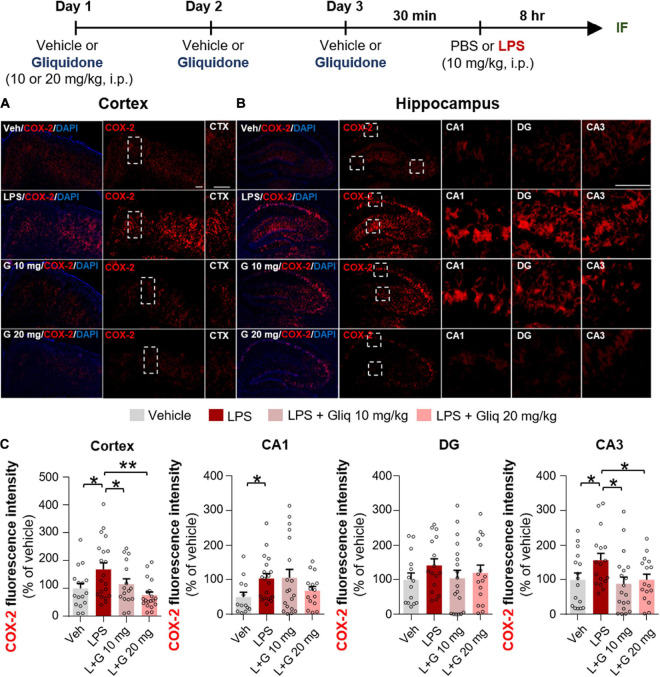
Gliquidone diminishes the LPS-induced elevation of COX-2 levels in the brain in C57BL6/N mice. **(A,B)** Immunofluorescence staining of COX-2 expression in brain slices from mice treated as shown at the top of the figure. **(C)** Quantification of the results in **(A,B)** (*n* = 11–18 brain slices from 4 mice/group). *p < 0.05, **p < 0.01, Scale bar = 100 μM.

**FIGURE 4:**
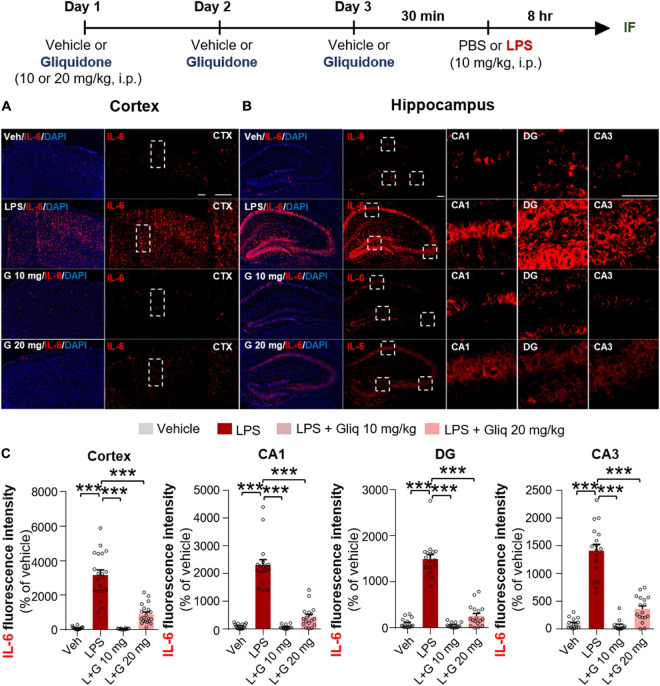
Gliquidone eliminates the LPS-induced elevation of IL-6 levels in the brain in C57BL6/N mice. **(A,B)** Immunofluorescence staining of IL-6 expression in brain slices from mice treated as shown at the top of the figure. **(C)** Quantification of the results in **(A,B)** (*n* = 12–20 brain slices from 4 mice/group). ***p < 0.001. Scale bar = 100 μM.

Since gliquidone downregulated LPS-induced micro- and astrogliosis in wild-type mice, we next investigated whether gliquidone modulates LPS-stimulated proinflammatory cytokine levels *in vivo*. Wild-type mice were treated as described above, and brain slices were subjected to IF staining with anti-COX-2 or anti-IL-6 antibodies. Treatment with 20 mg/kg gliquidone significantly diminished LPS-stimulated levels of COX-2 in the cortex and hippocampal CA3 region but not the hippocampal CA1 and DG regions (Figures 3A–C). At a dose of 10 mg/kg, gliquidone significantly decreased the LPS-evoked increase in COX-2 levels in the cortex and hippocampal CA3 region (Figures 3A–C). Moreover, injection of 10 or 20 mg/kg gliquidone following LPS administration markedly suppressed IL-6 levels in the cortex and hippocampus in wild-type mice, and the effects of 10 mg/kg gliquidone were significantly greater than those of 20 mg/kg gliquidone (Figures 4A–C). These findings suggest that gliquidone suppresses LPS-stimulated COX-2 and IL-6 levels in a model of neuroinflammatory disease.

### Gliquidone Inhibits Lipopolysaccharide-Stimulated NLRP3 Inflammasome Activation in Wild-Type Mice

**FIGURE 5:**
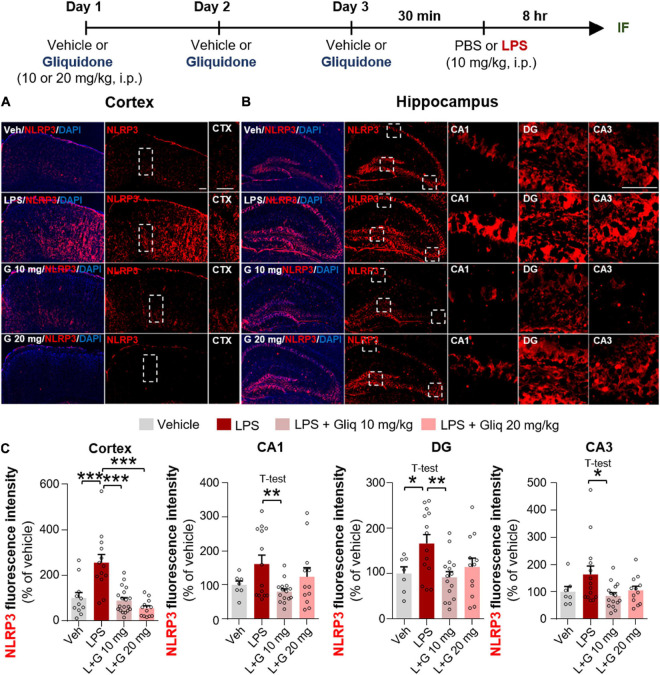
Gliquidone eliminates the LPS-mediated increase in NLRP3 inflammasome levels in the brain in C57BL6/N mice. **(A,B)** Immunofluorescence staining of NLRP3 in brain slices from mice treated as shown at the top of the figure. **(C)** Quantification of the results in **(A,B)** (*n* = 7–19 brain slices from 4 mice/group). *p < 0.05, **p < 0.01, ***p < 0.001. Scale bar = 100 μM.

The induction of proinflammatory cytokines by LPS is closely linked to microglia- and/or astrocyte-associated inflammation as well as inflammasome formation (Su et al., 2021; Zhao et al., 2021). Thus, we examined whether gliquidone affects LPS-induced NLRP3 inflammasome activation *in vivo*. Wild-type mice were treated as described above, and IF staining of brain slices was conducted with an anti-NLRP3 antibody. Injection of 10 mg/kg gliquidone significantly decreased LPS-induced NLRP3 activation in the cortex and hippocampus (Figures 5A–C), whereas 20 mg/kg gliquidone effectively diminished LPS-induced NLRP3 levels in the cortex but not the hippocampus (Figures 5A–C). These data indicate that gliquidone decreases LPS-stimulated NLRP3 inflammasome formation in the mouse brain.

### Gliquidone Downregulates Lipopolysaccharide-Induced Peripheral Proinflammatory Cytokine Levels in Wild-Type Mice

To investigate whether gliquidone alters peripheral proinflammatory cytokine levels *in vivo*, wild-type mice were treated as described above, followed by measurement of proinflammatory cytokine levels in peripheral blood by ELISA. Treatment of wild-type mice with 10 or 20 mg/kg gliquidone significantly abolished the LPS-induced increases in serum IL-1β and IL-6 levels but not TNF-α (Supplementary Figures 1A–C). These data indicate that gliquidone selectively alters peripheral proinflammatory cytokine levels in LPS-treated wild-type mice.

### Post- and Pretreatment of BV2 Microglial Cells With Gliquidone Abolishes Lipopolysaccharide-Induced Proinflammatory Cytokine mRNA Levels

**FIGURE 6:**
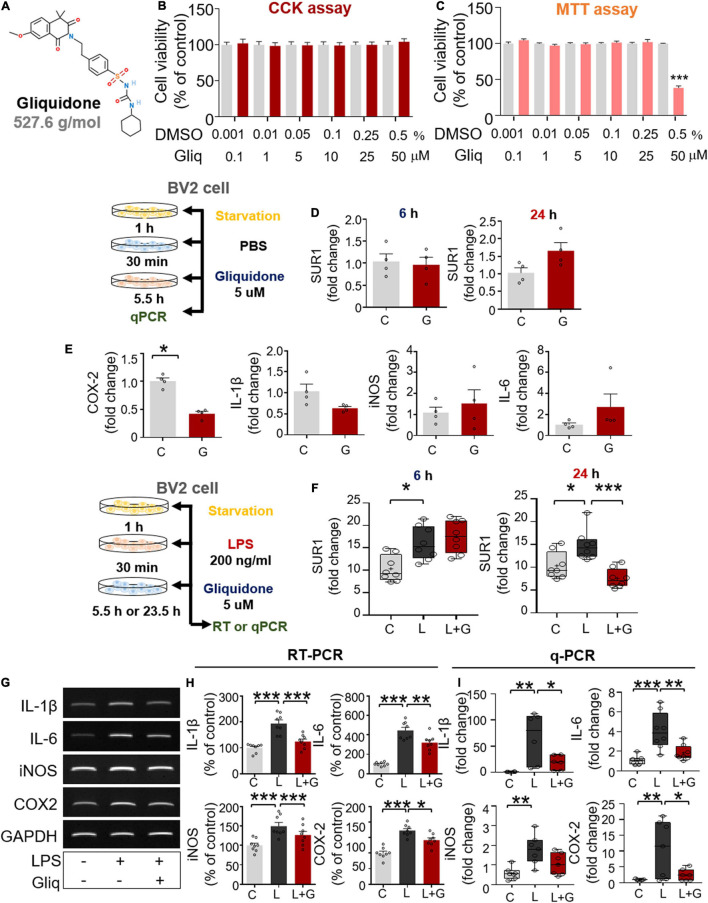
Post-treatment with gliquidone reduces LPS-stimulated proinflammatory cytokine mRNA levels in BV2 microglial cells. **(A)** Structure of gliquidone. **(B,C)** CCK and MTT assays were conducted after treating cells with gliquidone at the indicated doses or with vehicle for 6 or 24 h (CCK: 6 h, *n* = 16/dose; MTT: 24 h, *n* = 17/dose). **(D)** SUR1 mRNA levels in cells treated for 6 or 24 h with gliquidone alone or vehicle, as determined by q-PCR (*n* = 4/group). **(E)** Proinflammatory cytokine mRNA levels in cells treated with gliquidone alone or vehicle for 6 h, as determined by q-PCR (*n* = 4/group). **(F)** Real time-PCR (q-PCR) analysis of SUR1 mRNA levels in cells treated sequentially with LPS for 30 min and gliquidone for 5.5 or 23.5 h (*n* = 8/group). **(G,H)** RT-PCR analysis of proinflammatory cytokine levels in cells treated sequentially with LPS and gliquidone (*n* = 8/group). **(I)** Real time-PCR (q-PCR) analysis of proinflammatory cytokine levels in cells treated sequentially with LPS and gliquidone (IL-1β, *n* = 6/group; IL-6, *n* = 8/group; iNOS, *n* = 7/group; COX-2, *n* = 7/group). *p < 0.05, **p < 0.01, ***p < 0.001.

Since gliquidone regulates micro/astroglial activation, proinflammatory cytokine levels, and NLRP3 inflammasome activation in LPS-treated wild-type mice, we next verified the effects of gliquidone on LPS-mediated proinflammatory cytokine levels *in vitro.* We first assessed gliquidone cytotoxicity and induction of mitochondrial arrest using CCK or MTT assays in BV2 microglial cells. No cytotoxicity was observed in BV2 microglial cells after treatment with up to 50 μM gliquidone for 6 h (Figures 6A,B). In BV2 microglial cells treated for 24 h, gliquidone had no cytotoxicity up to 25 μM, but 50 μM gliquidone significantly diminished cell viability (Figure 6C). Based on these data, a gliquidone concentration of 5 μM, 10-fold lower than the concentration producing mitochondrial dysfunction, was used in subsequent experiments.

Gliquidone is a ligand of SUR1, which is part of the Kir6 channel (Wu et al., 2018), and thus we assessed the effects of gliquidone on SUR1 mRNA levels as well as proinflammatory cytokine levels by q-PCR. Treatment of BV2 microglial cells with gliquidone alone for 6 or 24 h did not alter SUR1 mRNA levels (Figure 6D). In addition, gliquidone alone reduced COX-2 mRNA levels but did not affect IL-1β, iNOS, and IL-6 mRNA levels in BV2 microglial cells (Figure 6E). These results indicate that 5 μM gliquidone itself selectively affects mRNA levels of the proinflammatory cytokine COX-2.

We then examined the effects of gliquidone on LPS-stimulated microglial SUR1 and proinflammatory cytokine mRNA levels in BV2 microglial cells. Cells were pretreated for 30 min with 200 ng/ml LPS or PBS and treated for 5.5 or 23.5 h with 5 μM gliquidone or vehicle (1% DMSO). Treatment with 5 μM gliquidone for 23.5 h but not 5.5 h significantly abolished the LPS-induced increase in SUR1 mRNA levels in BV2 microglial cells (Figure 6F). In addition, RT-PCR analysis showed that gliquidone post-treatment significantly reduced the LPS-induced increases in IL-1β, IL-6, iNOS, and COX-2 mRNA levels (Figures 6G,H). Confirming these results, q-PCR showed significant reductions in LPS-mediated IL-1β, IL-6, and COX-2 mRNA levels, with a trend toward decreased iNOS mRNA levels (Figure 6I). Moreover, we found that pretreatment with 5 μM gliquidone for 30 min significantly reduced the increases in IL-1β, IL-6, iNOS, and COX-2 mRNA levels in BV2 microglial cells induced by treatment with 200 ng/ml LPS for 5.5 h (Supplementary Figures 2A–C). Taken together, these results show that post- and pretreatment with gliquidone modulates LPS-induced microglial proinflammatory responses *in vitro*.

### Gliquidone Suppresses Lipopolysaccharide-Mediated Microglial ERK and STAT3/NF-κB Phosphorylation

**FIGURE 7:**
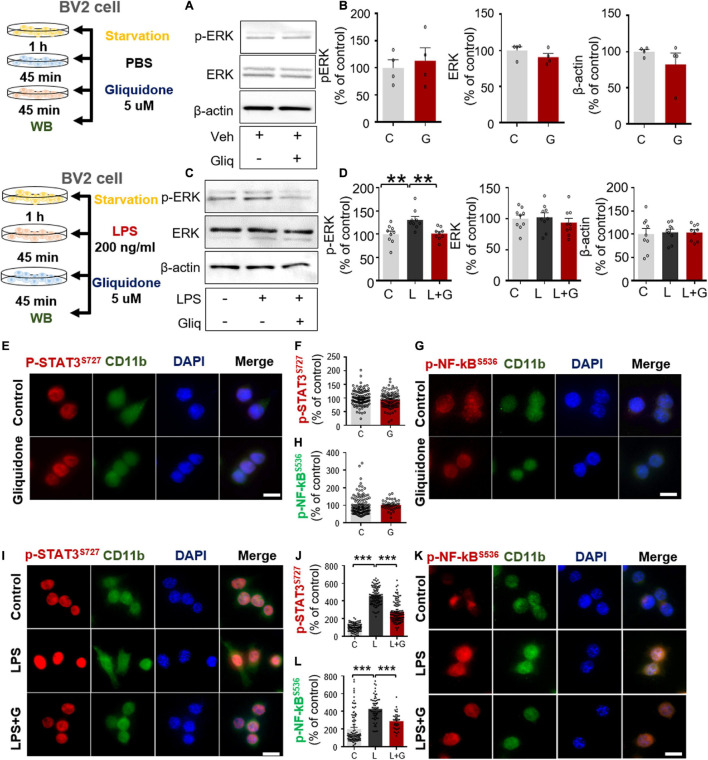
Gliquidone suppresses LPS-mediated ERK and STAT3/NF-κB phosphorylation in BV2 microglial cells. **(A,B)** Western blot analysis of p-ERK, ERK, and β-actin levels in cells treated with gliquidone alone or vehicle as shown (*n* = 4/group). **(C,D)** Western blot analysis of p-ERK, ERK, and β-actin levels in cells treated sequentially with LPS and gliquidone as indicated (*n* = 9/group). **(E–H)** Immunocytochemistry analysis of CD11b and p-STAT3^*S*727^ levels or CD11b and NF-κB^*S*536^ levels in cells treated with gliquidone or vehicle (p-STAT3^*S*727^: C, *n* = 112; gliquidone, *n* = 98; p-NK-kB^*S*536^: C, *n* = 149; gliquidone, *n* = 70). **(I–L)** Immunocytochemistry analysis of CD11b and p-STAT3^*S*727^ levels or CD11b and NF-κB^*S*536^ levels in cells treated sequentially with LPS and gliquidone (p-STAT3^*S*727^: C, *n* = 119; L, *n* = 100; LPS + gliquidone, *n* = 116; p-NK-kB^*S*536^: C, *n* = 101; L, *n* = 61; LPS + gliquidone, *n* = 37). **p < 0.01, ***p < 0.001. Scale bar = 20 μM.

In microglial cells, LPS stimulates TLR4 and ATP-sensitive K^+^ channels via ERK/STAT3 or NF-κB signaling (Rodriguez-Gomez et al., 2020). To examine whether gliquidone alone regulates ERK signaling mediated by ATP-sensitive K^+^ channels, BV2 microglial cells were treated sequentially with PBS for 45 min and 5 μM gliquidone or vehicle (1% DMSO) for 45 min. Subsequently, Western blotting was conducted with anti-p-ERK, anti-ERK, and anti-β-actin antibodies. We found that gliquidone alone did not alter p-ERK and total ERK levels in BV2 microglial cells (Figures 7A,B).

To examine whether gliquidone affects LPS-stimulated microglial ERK signaling, BV2 microglial cells treated with 200 ng/ml LPS or PBS for 45 min were post-treated with 5 μM gliquidone or vehicle (1% DMSO) for 45 min. Subsequently, Western blotting was conducted with anti-p-ERK, anti-ERK, and anti-β-actin antibodies. Gliquidone significantly decreased the LPS-induced increase in p-ERK levels but did not alter total ERK or β-actin levels (Figures 7C,D).

To assess the effects of gliquidone itself on nuclear STAT3 or NF-κB phosphorylation, BV2 microglial cells were sequentially treated with PBS for 30 min and 5 μM gliquidone or vehicle (1% DMSO) for 5.5 h, and ICC was performed with anti- p-STAT3^*s*727^ or anti-p-NF-κB antibodies. We found that gliquidone alone did not alter nuclear p-STAT3 and p-NF-κB levels in BV2 microglial cells (Figures 7E–H).

We then tested whether gliquidone alters LPS-mediated nuclear STAT3/NF-κB phosphorylation, BV2 microglial cells were sequentially treated with 200 ng/ml LPS or PBS for 30 min and 5 μM gliquidone or vehicle (1% DMSO) for 5.5 h. ICC assay demonstrated that gliquidone significantly reduced the LPS-mediated increases in nuclear STAT3 and NF-κB phosphorylation in BV2 microglial cells (Figures 7I–L). These results indicate that gliquidone modulates downstream ERK/STAT3 or NF-κB signaling regulated by TLR4 and ATP-sensitive K^+^ channels in microglial cells in response to neuroinflammation.

### Gliquidone Abolishes Lipopolysaccharide-Stimulated NLRP3 Inflammasome Activation in BV2 Microglial Cells

**FIGURE 8:**
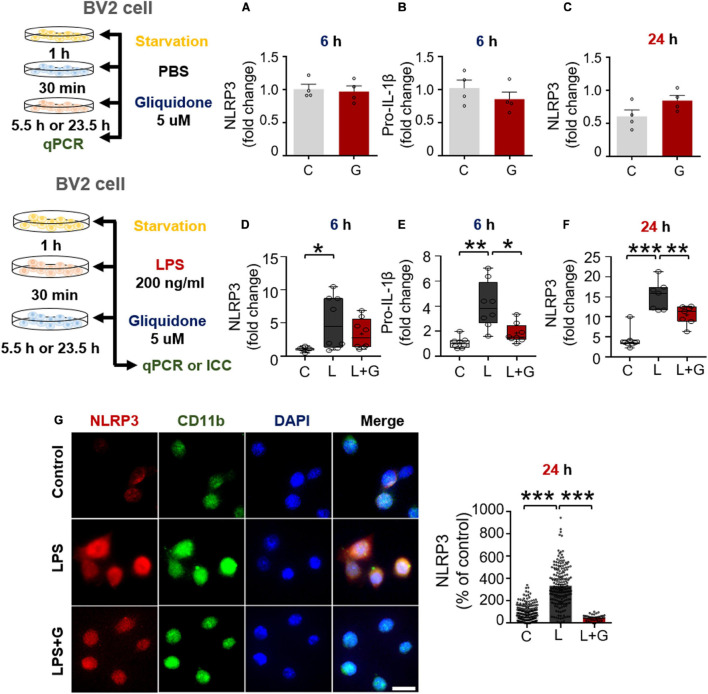
Gliquidone inhibits LPS-stimulated NLRP3 and pro-IL-1β in BV2 microglial cells. **(A–C)** Real time-PCR (q-PCR) analysis of NLRP3 and pro-IL-1β mRNA levels in cells treated with gliquidone alone or vehicle for 5.5 or 23.5 h (*n* = 4/group). **(D–F)** q-PCR analysis of NLRP3 and pro-IL-1β mRNA levels in cells treated sequentially with LPS and gliquidone for 6 or 24 h (6 h NLRP3 and pro-IL-1β, *n* = 8/group; 24 h NLRP3, *n* = 7/group). **(G,H)** Immunocytochemistry analysis of CD11b and p-NLRP3 levels in cells treated sequentially with LPS and gliquidone (C, *n* = 189; L, *n* = 198; LPS + gliquidone, *n* = 145). *p < 0.05, **p < 0.01, ***p < 0.001. Scale bar = 20 μM.

Since gliquidone downregulated LPS-evoked NLRP3 inflammasome formation in wild-type mice, we investigated whether gliquidone itself affects NLRP3 inflammasome activation. Treatment of BV2 microglial cells with PBS for 30 min followed by 5 μM gliquidone for 5.5 or 23.5 h did not alter NLRP3 and pro-IL-1β mRNA levels (Figures 8A–C).

We then investigated the effects of gliquidone on LPS-mediated microglial NLRP3 inflammasome activation. After treatment for 30 min with 200 ng/ml LPS or PBS, BV2 microglial cells were treated with 5 μM gliquidone or vehicle (1% DMSO) for 5.5 h or 23.5 h, and NLRP3/pro-IL-1β mRNA levels were detected by q-PCR. Post-treatment with gliquidone for 5.5 h significantly reduced LPS-enhanced pro-IL-1β mRNA levels, and a trend toward decreased NLRP3 mRNA levels was observed (Figures 8D,E). In addition, LPS-induced NLRP3 mRNA levels were markedly decreased by post-treatment with gliquidone for 23.5 h (Figure 8F). ICC analysis showed that gliquidone significantly downregulated LPS-induced microglial NLRP3 protein levels (Figures 8G,H). These data imply that gliquidone regulates the LPS-induced neuroinflammatory responses by decreasing NLRP3 inflammasome formation in BV2 microglial cells.

### Gliquidone Reduces Lipopolysaccharide-Evoked IL-1β and IL-6 mRNA Levels in Primary Astrocytes

**FIGURE 9:**
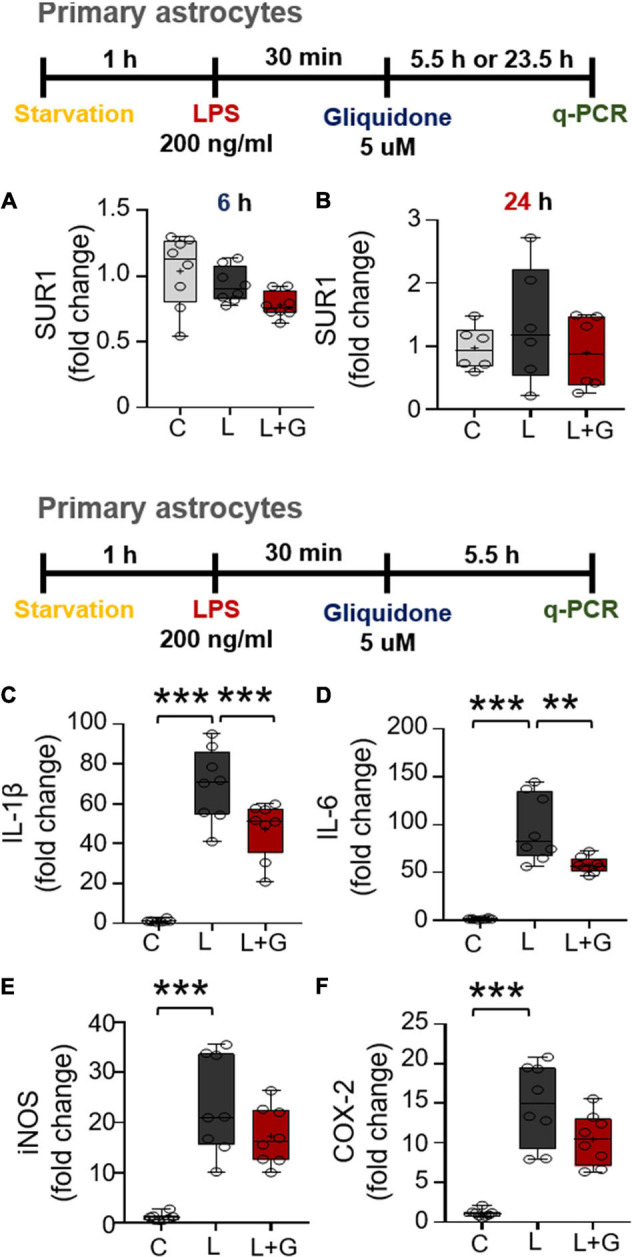
Post-treatment with gliquidone reduces LPS-stimulated IL-1β and IL-6 mRNA levels in primary astrocytes. **(A,B)** Real time-PCR (q-PCR) analysis of SUR1 mRNA levels in primary astrocytes treated sequentially with LPS and gliquidone as indicated (6 h, *n* = 8/group; 24 h, *n* = 6/group). **(C–F)** Real time-PCR (q-PCR) analysis of proinflammatory cytokine levels in primary astrocytes treated sequentially with LPS and gliquidone as indicated (*n* = 8/group). **p < 0.01, ***p < 0.001.

Since gliquidone downregulates LPS-mediated microglial neuroinflammatory responses in BV2 microglial cells, we next examined the effects of gliquidone on LPS-induced astrocytic neuroinflammatory responses. Mouse primary astrocytes were treated with 200 ng/ml LPS or PBS for 0.5 h and post-treated with 5 μM gliquidone or vehicle (1% DMSO) for 5.5 or 23.5 h. q-PCR analysis showed that gliquidone post-treatment for 5.5 or 23.5 h did not alter SUR1 mRNA levels (Figures 9A,B). In addition, gliquidone post-treatment significantly decreased LPS-stimulated IL-1β and IL-6 mRNA levels but did not alter iNOS and COX-2 mRNA levels (Figures 9C–F). These results suggest that gliquidone modulates proinflammatory cytokines less effectively in astrocytes than in microglia.

### Gliquidone Downregulates Lipopolysaccharide-Stimulated Nuclear STAT3 and NF-κB Phosphorylation in Primary Astrocytes

Given the significant reduction of downstream ERK signaling in BV2 microglial cells treated with gliquidone (Figure 7), we then investigated the effects of gliquidone on LPS-mediated astrocytic ERK signaling. Initially, we evaluated the purity of the primary astrocytes culture by measuring the GFAP/DAPI ratio, indicating an astrocyte purity of greater than 80% (Supplementary Figures 3A,B). Subsequently, mouse primary astrocytes were treated with 200 ng/ml LPS or PBS for 0.5 h followed by 5 μM gliquidone or vehicle (1% DMSO) for 5.5 h, and ICC was performed with an anti-p-ERK antibody. Gliquidone treatment did not affect LPS-evoked ERK phosphorylation in primary astrocytes (Supplementary Figures 3C,D).

**FIGURE 10:**
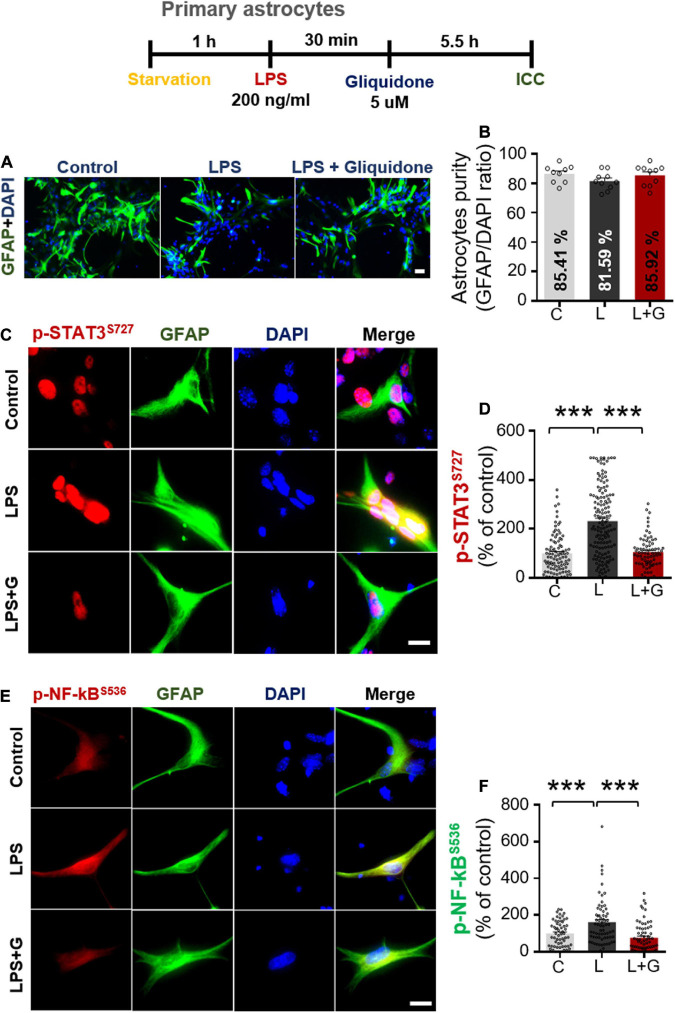
Gliquidone abolishes LPS-induced STAT3 and NF-κB phosphorylation in primary astrocytes. **(A,B)** Primary astrocytes were treated sequentially with LPS or PBS and with gliquidone (5 μM) or vehicle (1% DMSO) as indicated, and immunocytochemistry was performed with an anti-GFAP antibody. The purity of the primary astrocytes was measured by the GFAP/DAPI ratio (C, *n* = 1726; L, *n* = 876; LPS + gliquidone, *n* = 1527). **(C–F)** Immunocytochemistry analysis of GFAP and p-STAT3^*S*727^ or GFAP and p-NF-κB^*S*536^ expression in primary astrocytes treated sequentially with LPS and gliquidone (p-STAT3^*S*727^: C, *n* = 94; L, *n* = 145; LPS + gliquidone, *n* = 76; p-NF-κB^*S*536^: C, *n* = 59; L, *n* = 68; LPS + gliquidone, *n* = 75). ***p < 0.001, Scale bar = 20 μM.

Next, we examined the effects of gliquidone on LPS-induced astrocytic STAT3 or NF-κB phosphorylation in the nucleus. Again, we first confirmed that our primary astrocytes had a purity of greater than 80% (Figures 10A,B). Subsequent ICC analysis of cells treated as described above with anti-CD11b and anti-p-STAT3^*S*727^ or anti-CD11b and p-NF-κB^*S*536^ antibodies indicated that gliquidone significantly reduced the LPS-stimulated increases in nuclear STAT3 and NF-κB phosphorylation in primary astrocytes (Figures 10C–F). These results indicate that gliquidone modulates LPS-mediated astrocytic STAT3 or NF-κB signaling in a p-ERK-independent manner in response to neuroinflammation.

### Gliquidone Does Not Modulate Lipopolysaccharide-Induced NLRP3 Inflammasome Activation in Primary Astrocytes

Since gliquidone downregulated LPS-evoked proinflammatory cytokine levels and nuclear STAT3 and NF-κB phosphorylation in primary astrocytes, we further examined the effects of gliquidone on LPS-induced astrocytic NLRP3 inflammasome activation. Mouse primary astrocytes were treated with 200 ng/ml LPS or PBS for 0.5 h and post-treated with 5 μM gliquidone or vehicle (1% DMSO) for 5.5 h or 23.5 h, and NLRP3 and pro-IL-1β were detected by q-PCR or ICC. Gliquidone post-treatment for 5.5 h significantly diminished the LPS-induced increase in pro-IL-1β mRNA levels, whereas no changes in NLRP3 mRNA and protein levels were observed after gliquidone post-treatment for 5.5 or 23.5 h (Supplementary Figures 4A–D). These results indicate that gliquidone regulates LPS-mediated astrocytic proinflammatory cytokine levels by inhibiting STAT3/NF-κB signaling but does not modulate ERK signaling and NLRP3 inflammasome activation in primary astrocytes *in vitro*.

## Discussion

In this study, we demonstrated that gliquidone, a sulfonylurea drug that selectively binds sulfonylurea receptor subunit 1 (SUR1), significantly reduced LPS-induced microglial activation and COX-2 and IL-6 proinflammatory cytokine levels by inhibiting NLRP3 inflammasome activation in a model of neuroinflammatory disease, with smaller effects on astrogliosis. In LPS-treated BV2 microglial cells, gliquidone diminished LPS-induced microglial proinflammatory cytokine levels and downstream ERK/STAT3/NF-κB phosphorylation by suppressing NLRP3 inflammasome formation. By contrast, in primary astrocytes, gliquidone selectively abolished LPS-stimulated astrocytic proinflammatory cytokine levels by diminishing STAT3/NF-κB activation via NLRP3-independent mechanisms. The ability of gliquidone to alter LPS-mediated micro- and astroglial neuroinflammation *in vitro* and *in vivo* suggests that this drug could be useful for the prevention and/or treatment of neuroinflammation-associated diseases.

Gliquidone targets SUR1, an inhibitory and regulatory subunit of ATP-dependent K^+^ (K-ATP) channels (Ashcroft, 2005; Martin et al., 2017), leading to K-ATP channel closure. K-ATP channels are ubiquitously expressed in neurons and glia in several brain regions, including the cortex and hippocampus (McLarnon et al., 2001; Sun and Feng, 2013), and have been linked to inflammatory responses *in vitro* and *in vivo* (Zhou et al., 2008; Rodriguez et al., 2013; Du et al., 2018). For instance, levels of K-ATP channel components [e.g., inward-rectifier potassium subunit 6.1 (Kir 6.1), Kir 6.2, SUR1 and SUR2B] are increased in reactive microglia in neuroinflammation-linked brain pathologies (Ramonet et al., 2004; Ortega et al., 2012, 2013). Membrane and mitochondrial K-ATP channels regulate microglial and astroglia activation by controlling membrane electrical changes and mitochondrial ATP metabolism (Rodriguez et al., 2013). In cultured microglia derived from wild-type mice, LPS and interferon-γ (IFN- γ) co-treatment upregulates Kir 6.1 and Kir 6.2 (Virgili et al., 2011), and the K-ATP channel blocker glimepiride significantly reduces the expression of CD14 in BV2 microglial cells and primary microglia (Ingham et al., 2014). In addition, glibenclamide, a K-ATP channel blocker, alleviates neuroinflammation by inhibiting NLRP3 inflammasome activation in BV2 microglial cells and significantly reduces morphine-mediated Iba-1 fluorescence intensity in the spinal cord in mice (Qu et al., 2017). Moreover, K-ATP channels in astrocytes have been closely linked to neurodegeneration via mitophagy in a mouse model of PD, which increases NLRP3 inflammasome formation (Du et al., 2018). In the present study, we found that exposure to the K-ATP channel antagonist gliquidone significantly decreased LPS-mediated microglial activation and hypertrophy in the brains of wild-type mice (Figure 1). In addition, gliquidone significantly decreased LPS-mediated astrocyte activation and the number of GFAP-positive cells in the cortex but not the hippocampus (Figure 2). Taken together with the literature, our findings suggest that gliquidone specifically modulates microglial activation rather than astrogliosis by regulating K-ATP channel signaling in this LPS-induced model of neuroinflammatory disease.

K-ATP channel blockers influence proinflammatory cytokine mRNA and/or protein levels and release (Nakamura et al., 2015; Qu et al., 2017; Du et al., 2019). For example, glibenclamide significantly suppresses LPS-induced plasma IL-6 and TNF-α levels in *ex vivo* endotoxemia mice (Schmid et al., 2011). Daily administration of another sulfonylurea K-ATP channel blocker, gliclazide, significantly decreases serum TNF-α levels in albino LACA mice (Mourya et al., 2018). In the present study, gliquidone significantly suppressed LPS-induced COX-2 and IL-6 proinflammatory cytokine levels and peripheral inflammation in wild-type mice (Figures 3, 4 and Supplementary Figure 1). Moreover, gliquidone significantly downregulated LPS-mediated NLRP3 levels in wild-type mice (Figure 5). These data indicate that gliquidone and other K-ATP channel inhibitors can modulate proinflammatory cytokine expression as well as peripheral inflammation by altering NLRP3 inflammasome formation *in vivo*.

An important finding of this study is that gliquidone treatment significantly reduced LPS-mediated Iba-1, COX-2, IL-6, and NLRP3 levels in the cortex and/or hippocampal CA1/CA3 regions in wild-type mice but did not alter LPS-stimulated Iba-1 and COX-2 levels in the DG region (Figures 1, 3). However, this raises the question as to how does gliquidone differentially modulate the induction of Iba-1, COX-2, IL-6, and NLRP3 by LPS according to brain region? It is possible that the receptor subunit (i.e., SUR1 or Kir 6) and/or K-ATP channel targeted by gliquidone is differentially expressed in a brain region-specific manner (cortex, hippocampus CA1 vs. DG regions), resulting in differences in the effects of gliquidone on LPS-induced Iba-1, COX-2, IL-6, and NLRP3 levels between the cortex and hippocampus in wild-type mice. Another possibility is that a 3-day duration of daily injections was not sufficient to influence LPS-stimulated Iba-1, COX-2, IL-6, and NLRP3 levels in the hippocampus (CA1 vs. DG region). Future work, will need to examine whether a differential expression of the target receptor subunit (SUR1 and Kir6) and/or K-ATP channel exists between the cortex and hippocampal CA1 vs. DG region and if this alters LPS-induced proinflammatory responses in the presence and absence of gliquidone. Furthermore, the effect of increasing the duration of gliquidone treatment (i.e., daily for 7 or 14 days) upon LPS-evoked neuroinflammation in the cortex and hippocampus should be examined and compared with 3 days injection protocol.

K-ATP channel blockers have been previously shown to modulate proinflammatory cytokine expression *in vitro*. For instance, the sulfonylurea K-ATP channel blocker glimepiride significantly reduced the LPS-induced increase in TNF-α expression in RAW 264 macrophage cells (Ingham et al., 2014). In BV2 microglial cells, glibenclamide significantly suppressed morphine- and LPS-induced proinflammatory cytokine levels (Qu et al., 2017; Xu et al., 2017). Gliquidone is a second-generation K-ATP blocker with few side effects, but its ability to modulate LPS-induced neuroinflammatory responses and its mechanism of action have not been investigated. Here, we found that either pre- or post-treatment with gliquidone significantly reduced LPS-induced proinflammatory cytokine expression in BV2 microglial cells, which express the K-ATP channel subunits Kir 6.1 and Kir 6.2 (Rodriguez et al., 2013; Du et al., 2018) (Figure 6 and Supplementary Figure 2). In primary astrocytes, gliquidone significantly diminished LPS-induced proinflammatory cytokine levels (Figure 9), suggesting that gliquidone modulates the target receptor itself and that blockade of K-ATPs is critical for proinflammatory cytokine production in microglia and astrocytes. In future work, we will perform epigenetic knockdown or inhibit individual K-ATP channel subunits (i.e., SUR1, SUR2B, Kir 6.1, Kir 6.2) to examine whether gliquidone alters LPS-mediated proinflammatory cytokine release by regulating SUR1 specifically or all four K-ATP subunits. Overall, our findings suggest that K-ATP channel modulation by gliquidone reduces LPS-induced proinflammatory cytokine release from glial cells.

Lipopolysaccharide binds TLR4 to stimulate TLR4-linked proinflammatory cytokine production and transcription factor activation in microglia and astrocytes (Park and Lee, 2013; Molteni et al., 2016). Under normal conditions, K-ATP channels regulate Ca^2+^ influx, and increased Ca^2+^ conductance activates TLR4-associated MAPK signaling in microglia (Katz et al., 2006; Sharma and Ping, 2014). Indeed, several studies have demonstrated that TLR4- and K-ATP-linked MAPK/ERK signaling modulates proinflammatory cytokine expression (Xiao et al., 2002; Lin and Chai, 2008; Cargnello and Roux, 2011; Plociennikowska et al., 2015). For example, the first-generation K-ATP channel antagonist tolbutamide significantly inhibits ERK phosphorylation in U87 glioma cells, and this decrease is rescued by the K-ATP channel agonist diazoxide (Huang et al., 2009). In BV2 microglial cells, the K-ATP channel antagonist and SUR1 agonist glibenclamide significantly reduces LPS-induced MAPK signaling (e.g., p-ERK, p-JNK, and p-P38) in a dose-dependent manner (Xu et al., 2017). Here, we found that the anti-diabetic drug and second-generation K-ATP channel blocker gliquidone significantly reduced LPS-stimulated p-ERK levels in BV2 microglial cells but not in primary astrocytes (Figure 7 and Supplementary Figure 3), indicating that gliquidone affects TLR4- and K-ATP-associated ERK signaling in microglia. However, it is possible that gliquidone inhibits other MAPK signaling pathways to modulate LPS-induced proinflammatory responses; future experiments will address this possibility by using specific inhibitors and/or regulating gene expression levels (e.g., siRNA).

Given the effects of gliquidone on LPS-linked ERK phosphorylation in microglia, we investigated whether gliquidone modulates the transcription factor STAT3 and NF-κB, a downstream molecule that is crucial for promoting proinflammatory cytokine expression (Nam et al., 2018; Ryu et al., 2019). Recent studies have demonstrated links of K-ATP channels with STAT3 and NF-κB in the response to inflammation in the central nervous system (CNS) and peripheral nervous system (PNS). For instance, glibenclamide abolishes K-ATP channel opening-induced increases in rat hepatic p-STAT3 levels (Fouad et al., 2020). In human cerebrospinal fluid and cardiomyocytes, the K-ATP channel opener diazoxide and the mitochondrial K-ATP channel agonist rapamycin significantly increase STAT3 phosphorylation (Kishore et al., 2011; Das et al., 2012). Furthermore, morphine-induced impairment of the ATP/ADP ratio stimulates K-ATP channel-associated NF-κB activation by inducing NLRP3 inflammasome formation in microglia (Qu et al., 2017). In the present study, gliquidone significantly reduced LPS-mediated nuclear p-STAT3 and p-NF-κB levels in BV2 microglial cells and primary astrocytes (Figures 7, 10). Therefore, our findings and previous work suggest that gliquidone suppresses LPS-evoked STAT3/NF-κB activation by inhibiting K-ATP channels in microglia and primary astrocytes. It is possible that gliquidone affects LPS-mediated proinflammatory cytokine production in microglia and/or astrocytes by inhibiting another transcription factor, such as NFATc-1 (nuclear factor of activated T-cells, cytoplasmic 1), which is known to be involved in TLR4-mediated downstream signaling. Thus, future studies will examine whether gliquidone affects other LPS-associated MAPK signaling pathways and transcription factors linked to K-ATP channels.

Excessive activation of the NLRP3 inflammasome is a key contributor to pathogenesis in various neuroinflammatory responses. In addition, associations of NLRP3 with intracellular K efflux/concentration in microglia and astrocytes have recently been reported. For example, numerous NLRP3 inflammasome activators [i.e., danger-associated molecular patterns (DAMPs) such as silica and uric acid crystals and pathogen-associated molecular patterns (PAMPs) such as LPS and morphine] are known to induce potassium efflux (Qu et al., 2017; Yang et al., 2019). The K-ATP channel agonist LLME (lysosomotropic agent Leu-Leu-*O*-methyl ester) significantly increases intracellular potassium efflux in C57BL/6 mice and *Nlrp3*^–/–^ mice, which induces NLRP3 inflammasome activation independent of calcium signaling (Katsnelson et al., 2015). In addition, K efflux and NLRP3 inflammasome activation are significantly increased in LLME-treated bone marrow-derived dendritic cells, suggesting that NLRP3 is closely linked to K-ATP channel activation (Katsnelson et al., 2016). Reducing the intracellular K^+^ concentration is sufficient to activate NLRP3 inflammasome formation in nlrp3^–/–^ macrophages (Munoz-Planillo et al., 2013). Interestingly, the classic K-ATP channel blocker glibenclamide reduces morphine-induced neuroinflammation by inhibiting NLRP3 inflammasome activation in CD-1 mice (Qu et al., 2017). Consistent with these observations, we found that the second-generation K-ATP channel blocker gliquidone significantly inhibited LPS-mediated NLRP3 inflammasome activation in wild-type mice and BV2 microglial cells but not primary astrocytes (Figures 5, 8 and Supplementary Figure 4). These data indicate that gliquidone differentially regulates LPS-mediated NLRP3 inflammasome activation to alter neuroinflammatory responses in microglia and astrocytes. The underlying mechanism by which gliquidone influences signaling factors involved in NLRP3 inflammasome formation requires further investigation *in vivo* and *in vitro.*

## Conclusion

**FIGURE 11:**
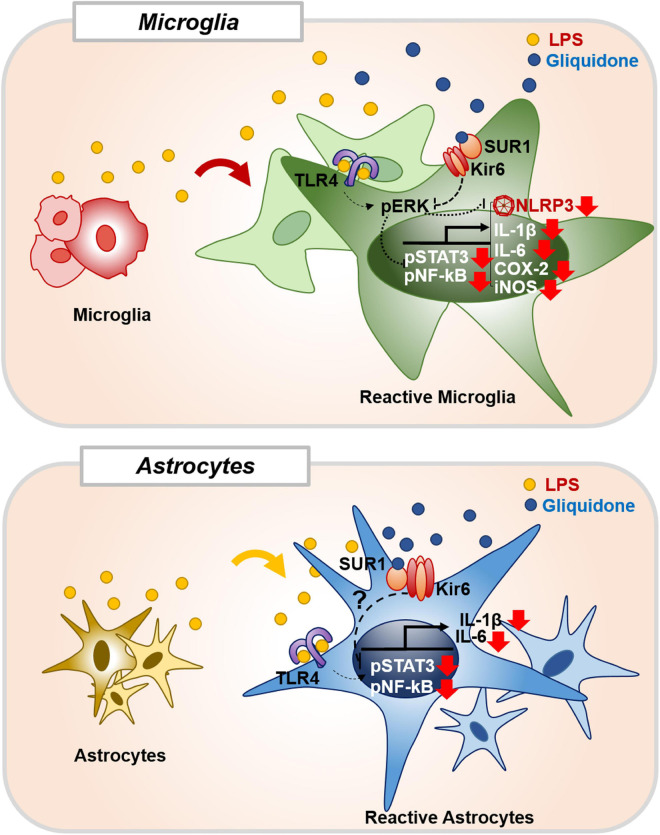
Gliquidone affects LPS-stimulated neuroinflammatory responses *in vitro* and *in vivo*. In BV2 microglial cells and wild-type mice, gliquidone reduces LPS-stimulated microglial proinflammatory cytokine expression by modulating ERK-associated STAT3/NF-κB signaling in an NLRP3-dependent manner. In primary astrocytes, gliquidone selectively downregulates LPS-induced astrocytic proinflammatory cytokine expression by decreasing STAT3/NF-κB phosphorylation via an NLRP3-independent pathway. Accordingly, gliquidone may have therapeutic potential for neuroinflammation-related neurodegenerative diseases.

In summary, gliquidone downregulates LPS-stimulated increases in microglial morphology and activation as well as proinflammatory cytokine IL-6 and COX-2 levels by decreasing NLRP3 inflammasome formation in wild-type mice. Compared with its effects on microglia, gliquidone has smaller effects on astrogliosis and astroglial hypertrophy in LPS-treated wild-type mice. In BV2 microglial cells, gliquidone suppresses LPS-induced proinflammatory cytokine levels by modulating ERK/STAT3/NF-κB phosphorylation through the inhibition of NLRP3 inflammasome activation. In primary astrocytes, gliquidone selectively affects LPS-mediated proinflammatory cytokine levels and reduces STAT3/NK-kB phosphorylation in an NLRP3-independent manner (Figure 11). These results suggest that gliquidone has anti-inflammatory effects by modulating LPS-induced micro- and astroglial neuroinflammation and could be a novel therapy for neuroinflammation-related neurodegenerative diseases.

## Data Availability Statement

The original contributions presented in the study are included in the article/Supplementary Material, further inquiries can be directed to the corresponding author.

## Ethics Statement

The animal study was reviewed and approved by Korea Brain Research Institute (KBRI, approval no. IACUC-19-00042).

## Author Contributions

JK and H-SH: study conception and design. J-HP and JK: acquisition of data. JK: preparation of figures and tables. JK, KS, SJM, KC, and H-SH: writing and editing of manuscript. All authors contributed to the article and approved the submitted version.

## Conflict of Interest

The authors declare that this study received funding from Whanin Pharm Co., Ltd. The funder was not involved in the study design, collection, analysis, interpretation of data or the writing of this article.

## Publisher’s Note

All claims expressed in this article are solely those of the authors and do not necessarily represent those of their affiliated organizations, or those of the publisher, the editors and the reviewers. Any product that may be evaluated in this article, or claim that may be made by its manufacturer, is not guaranteed or endorsed by the publisher.
