# Supplementary material for: The Anti-diabetic Drug Gliquidone Modulates Lipopolysaccharide-Mediated Microglial Neuroinflammatory Responses by Inhibiting the NLRP3 Inflammasome
Source: Front Aging Neurosci. 2021 Oct 29;13:754123. doi: 10.3389/fnagi.2021.754123 (PMC8587901; doi:10.3389/fnagi.2021.754123)
Supplement: Supplementary file 1 [file Data_Sheet_1.docx]

Supplementary Figures

**The anti-diabetic drug gliquidone modulates LPS-mediated microglial neuroinflammatory responses by inhibiting the NLRP3 inflammasome**

Jieun Kim^1^, Jin-Hee Park^1^, Keshvi Shah^1,3^, Scott John Mitchell^3^, Kwangwook Cho^3^, Hyang-Sook Hoe^1,2*^

^1^Neurogegnerative diseases group, Korea Brain Research Institute (KBRI), 61, Cheomdan-ro, Dong-gu, Daegu, Korea. 41068; ^2^Department of Brain & Cognitive Sciences, Daegu Gyeongbuk Institute of Science & Technology (DGIST), Daegu, Korea, 42988; ^3^UK-Dementia Research Institute at King’s College London, Department of Basic and Clinical Neuroscience, Institute of Psychiatry, Psychology and Neuroscience, King’s College London, SE5 9NU, UK

*Corresponding author

Hyang-Sook Hoe, Ph.D.: Department of Neural Development and Disease, Korea Brain Research Institute (KBRI), 61 Cheomdan-ro, Dong-gu, Daegu, Korea, 41068

E-mail: [sookhoe72@kbri.re.kr](mailto:sookhoe72@kbri.re.kr)

**
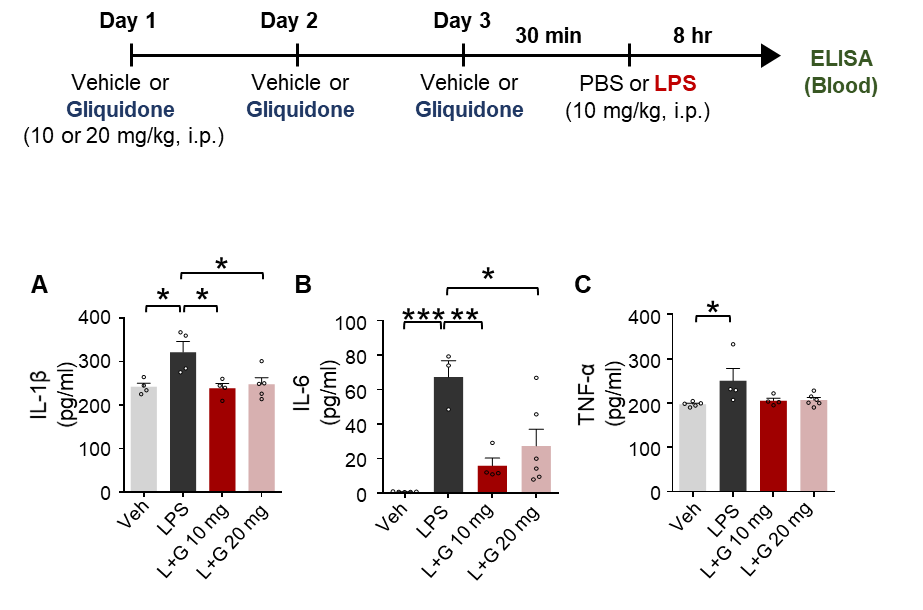
**

**Supplementary Figure 1**. Gliquidone suppresses LPS-induced peripheral proinflammatory cytokine levels in C57BL6/N mice. The *in vivo* experimental procedure for gliquidone and LPS injection is shown at the top of the figure. (A-C) ELISA of the proinflammatory cytokines IL-1β, IL-6, and TNF-α in mice treated as described in the experimental procedure (IL-1β: Veh, n = 4; LPS, n = 4; L+G 10 mg, n = 4; L+G 20 mg, n = 5; IL-6: Veh, n = 5; LPS, n = 3; L+G 10 mg, n = 4; L+G 20 mg, n = 6; TNF-α: Veh, n = 5; LPS, n = 4; L+G 10 mg, n = 4; L+G 20 mg, n = 6). *p < 0.05, **p < 0.01, ***p < 0.001.

**
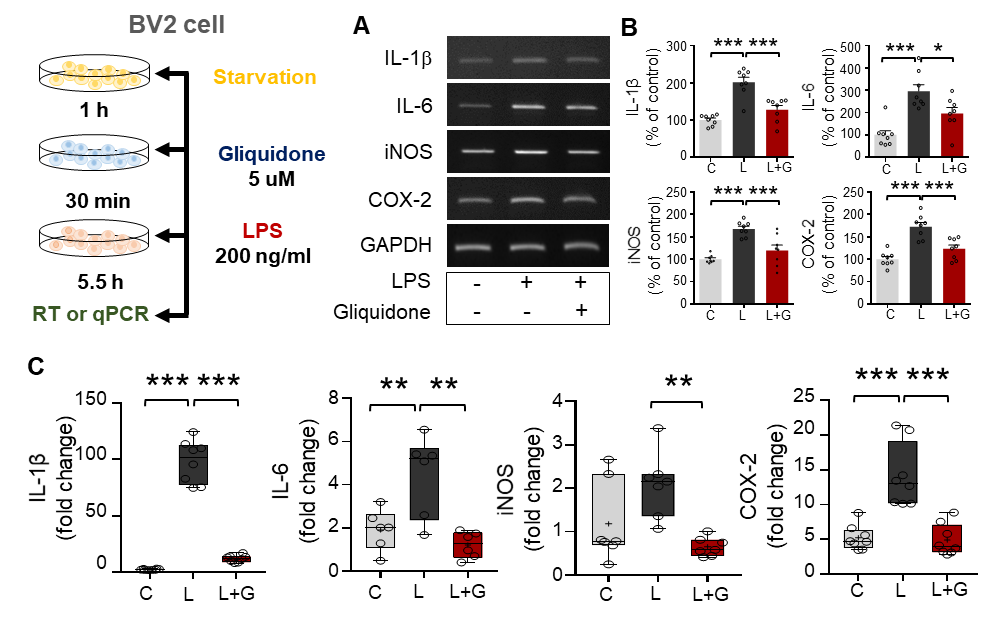
**

**Supplementary Figure 2**. Pretreatment with gliquidone abolishes LPS-stimulated IL-1β, IL-6, iNOS and COX-2 mRNA levels in BV2 microglial cells. (A-B) RT-PCR analysis of proinflammatory cytokine mRNA levels in cells treated sequentially with gliquidone and LPS as indicated (n = 8/group). (C) Q-PCR analysis of proinflammatory cytokine mRNA levels in cells treated sequentially with gliquidone and LPS (IL-1β, n = 8/group; IL-6, n = 6/group; iNOS, n = 7/group; COX-2, n = 8/group). *p < 0.05, **p < 0.01, ***p < 0.001.

**
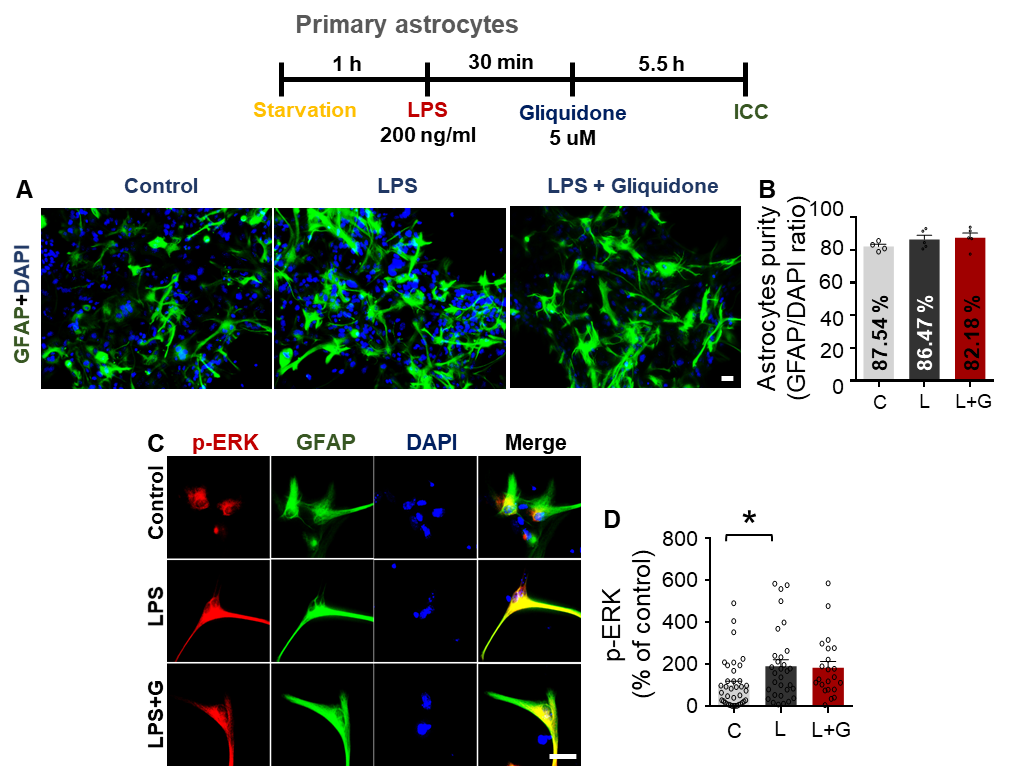
**

**Supplementary Figure 3**. Gliquidone does not alter LPS-stimulated astroglial ERK phosphorylation. (A-B) Primary astrocytes were treated sequentially with LPS or PBS and with gliquidone (5 μM) or vehicle (1% DMSO) as indicated, and immunocytochemistry was performed with an anti-GFAP antibody. The purity of the primary astrocytes was measured by the GFAP/DAPI ratio (C, n = 586; L, n = 839; LPS + gliquidone, n = 675). (C) Immunocytochemistry analysis of GFAP and p-ERK expression in primary astrocytes treated sequentially with LPS and gliquidone as indicated. (D) Quantification of the results in C (C, n = 38; L, n = 31; LPS + gliquidone, n = 22). *p < 0.05, Scale bar = 20 μM.


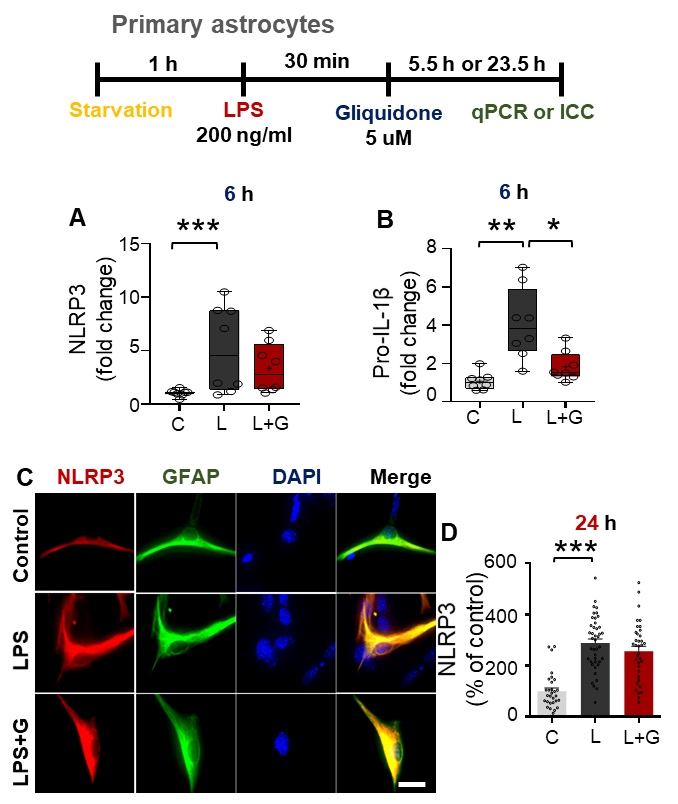


**Supplementary Figure 4**. Gliquidone does not alter LPS-stimulated astroglial NLRP3 and pro-IL-1β levels. (A-B) Q-PCR analysis of NLRP3 and pro-IL-1β mRNA levels in primary astrocytes treated sequentially with LPS and gliquidone as indicated (n = 8/group). (C) Immunocytochemistry analysis of GFAP and NLRP3 expression in primary astrocytes treated as indicated. (D) Quantification of the results in C. (C, n = 30; L, n = 42; LPS + gliquidone, n = 36). *p < 0.05, ***p < 0.001. Scale bar = 20 μM.
